# The calming effect of maternal carrying in different mammalian species

**DOI:** 10.3389/fpsyg.2015.00445

**Published:** 2015-04-16

**Authors:** Gianluca Esposito, Peipei Setoh, Sachine Yoshida, Kumi O. Kuroda

**Affiliations:** ^1^Affiliative Behavior and Physiology Laboratory, Department of Psychology and Cognitive Science, University of Trento, Rovereto, Italy; ^2^Division of Psychology, Nanyang Technological University, Singapore, Singapore; ^3^Faculty of Medicine, Toho University, Tokyo, Japan; ^4^Japan Science and Technology Agency, Precursory Research for Embryonic Science and Technology, Saitama, Japan; ^5^Unit for Affiliative Social Behavior Unit, RIKEN Brain Science Institute, Saitama, Japan

**Keywords:** maternal carrying, mother–infant interaction, mother–child relations, mother–infant bonding, transport response, attachment

## Abstract

Attachment theory postulates that mothers and their infants possess some basic physiological mechanisms that favor their dyadic interaction and bonding. Many studies have focused on the maternal physiological mechanisms that promote attachment (e.g., mothers’ automatic responses to infant faces and/or cries), and relatively less have examined infant physiology. Thus, the physiological mechanisms regulating infant bonding behaviors remain largely undefined. This review elucidates some of the neurobiological mechanisms governing social bonding and cooperation in humans by focusing on maternal carrying and its beneficial effect on mother–infant interaction in mammalian species (e.g., in humans, big cats, and rodents). These studies show that infants have a specific calming response to maternal carrying. A human infant carried by his/her walking mother exhibits a rapid heart rate decrease, and immediately stops voluntary movement and crying compared to when he/she is held in a sitting position. Furthermore, strikingly similar responses were identified in mouse rodents, who exhibit immobility, diminished ultra-sonic vocalizations and heart rate. In general, the studies described in the current review demonstrate the calming effect of maternal carrying to be comprised of a complex set of behavioral and physiological components, each of which has a specific postnatal time window and is orchestrated in a well-matched manner with the maturation of the infants. Such reactions could have been evolutionarily adaptive in mammalian mother–infant interactions. The findings have implications for parenting practices in developmentally normal populations. In addition, we propose that infants’ physiological response may be useful in clinical assessments as we discuss possible implications on early screening for child psychopathology (e.g., autism spectrum disorders and perinatal brain disorders).

## Introduction

In an early formalization of the Attachment Theory, Bowlby (1958) described five patterns of behavior (sucking, clinging, following, crying, and smiling) as automatic responses that promote caregiver–infant bonding. Later, in a more definitive formalization of the Attachment Theory, Bowlby (1969) postulated that, at some stage in the development of the behavioral system responsible for attachment, proximity to mother becomes a set-goal. In this theorization, the five patterns are still held to be of great importance because they represent a way to maintain maternal proximity. However, Bowlby (1969) describes how other behaviors may also serve the same function of maintaining maternal proximity. For example, the infant cooperation toward maternal carrying (Esposito et al., 2013) may increase maternal proximity and ultimately mother–infant bonding. In this review, we will summarize studies which have been conducted since the 1950s that have focused on infant postural response to maternal carrying across mammalian species (from rodents, to big cats and humans), and describe the recent findings on infant physiological responses to maternal carrying in human infants and in mice.

## The Calming Effects of Maternal Carrying in Humans

**FIGURE 1:**
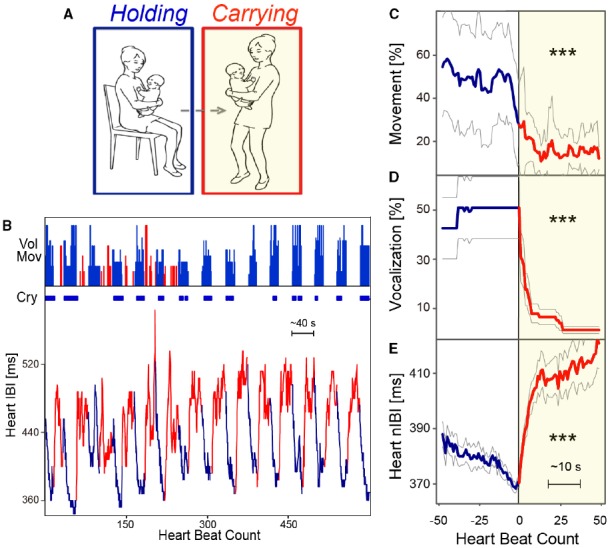
**Carrying-induced calming responses in human infants. (A)** Behavioral task of holding (blue) and carrying (red with yellow background) by the mother–infant dyad. **(B)** An example of the task consisting of repetition of holding and carrying. Each condition lasted approximately 20 s. Amount of voluntary movement, presence of crying, and the interbeat interval (IBI) of the infant are presented. **(C–E)** Time course of voluntary movements **(C)**, crying **(D)**, and the normalized IBI (nIBI) **(E)** of the holding-carrying transition of 12 human infants under 6 months of age. Extracted and reproduced from Esposito et al. (2013). ***p < 0.001.

Human caregivers commonly soothe babies by carrying them in our arms, in a sling, or in a stroller while walking and/or rocking them. However, whether infant carrying has a calming effect has been controversial (Hunziker and Barr, 1986; Walker and Menahem, 1994; St. James-Roberts et al., 1995). In a randomized controlled trial on 99 mother–infant dyads, Hunziker and Barr (1986) found that the typical amount of crying could be reduced by supplemental carrying, that is, increased carrying throughout the day in addition to that which occurs during feeding and in response to crying. They concluded that supplemental carrying modifies “normal” crying by reducing the duration and altered the typical pattern of crying and fussing in the first 3 months of life. Furthermore, it was speculated that the relative lack of carrying in our society may predispose normal infants to crying and colic (Hunziker and Barr, 1986). However, two subsequent studies did not find a beneficial effect of supplementary carrying. Walker and Menahem (1994) studied the role of supplementary carrying. Forty-three typically developing infants were randomly assigned to an intervention or control group. Infants in the intervention group were carried by their mothers in a soft ventral baby sling for at least 2 h a day, and their crying and behavior patterns were documented in a 24-h diary at 1, 2, 4, 6, and 8 weeks of age. There was no statistical difference between the two conditions (Walker and Menahem, 1994). This was corroborated by St. James-Roberts et al. (1995) who also did not find any differences in amounts of crying and fussing. These findings have been taken to suggest that supplementary carrying cannot be used as a primary, preventative intervention to reduce infant crying (St. James-Roberts et al., 1995). However, these three studies measured the total amount of crying and “supplementary” carrying by relying on parental diaries, rather than direct observation, and the relationship found between crying and carrying was analyzed through correlations in hour-order time bins. No distinction was made in the parental reports between mobile carrying and simple holding without movement. In more recent works from our group (e.g., Esposito et al., 2013; see also Cadena, 2013; Gammie, 2013), we have contributed to this debate, suggesting three main experimental changes (compared to the previous studies). First, we use direct, real-time measures of both mothers’ actions and infants’ responses. Second, we use multiple methods for analyzing infant physiological and psychological responses (including electrocardiogram and audio/video monitors). Third, we define “carrying” (which includes holding and walking) in a way that clearly distinguishes it from just stationary holding. With these advances, we find a clear and significant decrease in infant voluntary movement, heart rate, and crying when infants are carried rather than held in a sitting position (see Figure 1).

## The Calming Effects of Maternal Carrying in Other Mammals

In many mammalian species with altricial young, the mother carries her offspring by mouth to transport them for various reasons, such as to conceal them while she forages for food, or to move nests, or away from danger. In a variety of mammalian species such as cats (Schaller, 1972), rodents (Eibl-Eibesfeldt, 1951; Zippelius, 1971), and primate (Sauer, 1967; Wilson et al., 2000, 2008), infants assume a passive and compact posture with their hind legs drawn up while being carried. This postural regulation has been studied experimentally in laboratory rats as “transport response” (Brewster and Leon, 1980; Wilson et al., 1984). However, until recently, there has not been comparative studies of this phenomenon in mammalian species, nor detailed investigations into quantitative measurements for immobilization during carrying and physiological aspects of the phenomenon.

**FIGURE 2:**
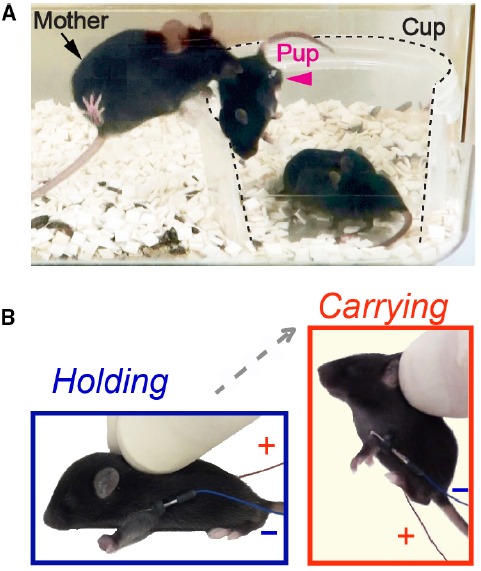
**Carrying-induced calming responses in mouse pups. (A)** The behavioral task of maternal rescue, in which a laboratory mouse mother (arrow) rescued her pup (arrowhead) from a transparent plastic cup fixed in the home cage. **(B)** Manual holding (blue) and carrying (red) of a mouse pup with two electrodes (+ and –) for ECG recording. Extracted and reproduced from Esposito et al. (2013).

New studies have investigated maternal retrieval behavior and pup’s calming response from a different perspective, examining them as a mutually dependent, dynamic process (Esposito et al., 2012, 2013; Yoshida et al., 2013). To explore this dyadic interaction in detail, we created a new experimental task of “maternal rescue of pups from a cup” (see Figure 2A), which is meant to mimic a challenging situation in the wild. Pups were placed in a plastic cup and their mother had to retrieve the pups from a cup back to the nest. Employing the “maternal rescue of pups from a cup” method, and the experimenter’s manual carrying procedure (by holding the small amount of skin at the nape of the neck, mimicking the maternal oral grasp; see Figure 2B), we examined the mouse pups’ response to maternal carrying, and found that pups, similar to human infants, immediately show a reduction in crying, body movement and heart rate during carrying (Esposito et al., 2013; see also Bizzego et al., 2014, for data modeling infants’ heart rate variability during maternal carrying). Therefore, in both mouse pups and human infants, carrying induced a similar calming responses in the offspring, even though maternal carrying methods differed, We also investigated the upstream and downstream neural systems that regulate the pups’ calming response using pharmacologic and genetic interventions (Esposito et al., 2013). We found that somatosensory and proprioceptive inputs are necessary to elicit the response. Furthermore, parasympathetic and cerebellar functions mediate cardiac and motor output respectively. Pups’ loss of the postural regulation hindered the effectiveness of their mother’s rescue, suggesting a functional significance for the identified calming response. We postulate that the calming response supports an affiliative mother–infant relationship, and increases the infant’s chance of survival during emergencies.

Following up on our findings, we subsequently redescribed the pup’s calming response composed of several behavioral and physiological changes as “Transport Response” (with capitalization showing respect for the previous study by Brewster and Leon, 1980) by examining how and when each behavioral and physiological change emerge in preweaned laboratory mice (Yoshida et al., 2013). Our ontogenic analyses revealed that mouse Transport Response is confined within a specific postnatal time window subdivided into four phases. In the first phase, which takes place approximately during the first postnatal week, the pups only show a reduction in ultrasonic vocalization without clear immobilization and postural changes. In the second phase, which takes place approximately during the second postnatal week, there is greater passivity, which includes heart rate reduction, robust immobilization and a relative insensitivity to the environment. In the third phase, which lasts from the end of the second postnatal week to the first few days of the third week, the pups weigh about a quarter of what their mothers weigh. Passive immobilization along with active postural regulations, including limb ventroflexion and body compaction, may be required for the mother to carry her pup efficiently. In addition, apparent analgesia was observed during the Transport Response. One of the possible function of the analgesia is that, with increased pain tolerance, pups are able to maintain calmness and remain immobilized, which aids in fast relocation via maternal oral transport during emergencies such as nest destruction (Brewster and Leon, 1980). During the last phase, which corresponds to the remainder of the third postnatal week, the pups’ mobility became more mature and their eyelids are fully opened, so that they were able to visually orient and travel by themselves. The pups’ immobilization response declines and reach the adult levels by the time of weaning. Correspondingly, the mice mothers refrain from orally retrieving its pups.

Taken together, the data the mouse Transport Response changes in accordance with the physical maturation of the pup. The Transport Response is a filial reaction to maternal carrying which ultimately increases the probability of the pups’ own survival.

## Evolutionary Basis of Infant Calming Responses to Maternal Carrying

Maternal touch and rhythmic rocking (vestibular-proprioceptive stimulation) is calming to both human infants (Vrugt and Pederson, 1973; Gray et al., 2000) and mouse pups (Esposito et al., 2013; Yoshida et al., 2013). Indeed animal studies find that the tactile sensation from maternal grasp and proprioception are required to elicit the carrying-induced calming responses. Extrapolating from this, walking for humans may be the most ethologically similar stimulation as it contributes both tactile sensory input and ambulatory motion, which may render walking more effective in calming infants than other kinds of rhythmic motion such as mechanical rocking.

Therefore, the infant calming responses may have the evolutionary function of increasing the survival probability of the infant in cases of emergency escape by the mother–infant dyad and ultimately work to support the mother–infant relationship. Conservation of this calming response in altricial mammalian species supports the adaptive value of this behavior in mother–infant relationship and, as a consequence, infant survival (Eibl-Eibesfeldt, 1951; Sauer, 1967; Brewster and Leon, 1980).

## Implications for Parental Practices

The identified effects of carrying on parasympathetic activation and cry reduction are significant and robust, so a brief period of carrying could be effective in soothing infants who are distressed by transient aggravations such as vaccinations or frightening noises. However, because the calming effect is only limited to the period of ambulatory carrying, the infant may resume crying if the underlying aggravation remains after the carrying ends, like hunger or chronic pain. A scientific understanding of infants’ physiological response could prevent parents from overreacting to infants’ crying. Such understanding would be beneficial to parents by reducing frustration, because unsoothable crying is a major risk factor for child abuse (Reijneveld et al., 2004).

## Implications for Developmental Psychopathology

Understanding how infants respond physiologically to caregiver holding may have useful applications in the field of child psychopathology as an assessment tool. Very early malfunctioning of the infant responses to maternal carrying can potentially be used as an early biomarker of autism spectrum disorders (ASDs), and may also provide an opportunity for an early estimation of the prognosis for infants with perinatal brain disorders (PBDs, i.e., Cerebral Palsy).

### Atypical Response to Maternal Carrying as Early Biomarker of Autism Spectrum Disorders

Autism spectrum disorders is a severe lifelong developmental disorder with a very high prevalence (affects 1 out of 88 infants) and is characterized by difficulties in social interaction and communication, as well as by repetitive behaviors and restricted interests. Many studies have highlighted that early diagnosis can lead to a substantial improvement in the life conditions of people with ASDs (Yirmiya and Charman, 2010). For this reason, the search for early biomarkers of the syndrome is extremely important, and is made even more pertinent by the consideration that current diagnosis methods are based on behavioral observation and can be reliable only when the child has at least 18 months of age. An interesting observation related to maternal carrying is that parents of infants with ASD report that their infants have difficulties in adjusting their body to being held (Kanner, 1943; Teitelbaum et al., 1998, 2004; Esposito et al., 2009, 2011; Esposito, 2011; Esposito and Paşca, 2013), and their parents sometimes make remarks such as, “I feel as if I were holding a stone or a sac of flour, not a baby.” Moreover, the neural systems regulating the responses to maternal carrying have also been implicated in the neuropathology of ASDs, such as cerebellar structure (Bauman and Kemper, 2005) and in sensory integration (Minshew et al., 2004). Thus it would be an interesting future research direction to examine whether the atypicality in the responses to maternal carrying may be an early biomarker of ASD.

### Autonomic Response to Maternal Carrying as Early Prognosis Estimator of Perinatal Brain Diseases

Perinatal brain diseases pertains to the period immediately before and after birth, and they may have very different causes (i.e., from infection or problems during parturition). PBDs are a very heterogeneous group of conditions that may cause various disabilities in development. The long-term prognosis of PBDs are difficult to predict, and exploration of good estimator of the prognosis of the disease would be of great medical implications. To aid in this endeavor, we plan to test whether the calming response to maternal carrying may be used as a measure of infants’ sensory and/or autonomic reactivity, and may somehow improve the prognosis of acquired PBD. We hypothesize that children with autonomic nervous system dysregulation, which has a more severe prognosis, may show atypicality in the autonomic response (specifically the parasympathetic modulation of Heart Rate) to maternal carrying from early postnatal months.

## Conclusion

Attachment theory postulates that mothers and their infants possess some basic physiological mechanisms that favor their caregiver–infant dyadic interaction and bonding. In this review, we describe studies on infant calming responses to maternal carrying across different species of mammals. In line with the predictions of the Attachment theory, we demonstrate that carrying-induced calming consist of a canonical set of behavioral and physiological responses in altricial mammalian infants which functions to facilitate an effective mother–infant relationship. Taking the lead from John Bowlby’s work, we believe that integrating studies from both psychological and physiological domains will provide better understanding into behaviors that create a manageble and rewarding caregiver–infant interaction, which may ultimately contribute to a good attachment relationship.

### Conflict of Interest Statement

The authors declare that the research was conducted in the absence of any commercial or financial relationships that could be construed as a potential conflict of interest.
